# Pleiotropic effects of signal peptide peptidase A (*sppA*) gene deletion on membrane homeostasis, gliding motility, and virulence in *Flavobacterium columnare*

**DOI:** 10.3389/fvets.2026.1784124

**Published:** 2026-05-22

**Authors:** Ruoxi Zhu, Liang Zhong, Yuying Xun, Shucheng Zheng, Yongtao Zhu, Wenlong Cai

**Affiliations:** 1 Department of Infectious Disease and Public Health, Jockey Club College of Veterinary Medicine and Life Science, City University of Hong Kong, Hong Kong, Hong Kong SAR, China; 2 Department of Biosciences and Bioinformatics, School of Science, Xi’an Jiaotong-Liverpool University, Suzhou, Jiangsu, China; 3 Wisdom Lake Academy of Pharmacy, Xi’an Jiaotong-Liverpool University, Suzhou, Jiangsu, China; 4 Key Laboratory of Fishery Drug Development, Ministry of Agriculture and Rural Affairs, Guangdong Provincial Key Laboratory of Aquatic Animal Immunology and Sustainable Aquaculture, Pearl River Fisheries Research Institute, Chinese Academy of Fishery Sciences, Guangzhou, Guangdong, China

**Keywords:** aquatic pathogen, bacterial virulence, columnaris disease, fish health, outer membrane vesicle, SppA, sustainable aquaculture

## Abstract

Columnaris disease, caused by *Flavobacterium columnare*, represents one of the most economically devastating bacterial infections in global freshwater aquaculture. Despite its significant impact, the molecular mechanisms underlying *F. columnare* pathogenesis remain largely unexplored. Signal peptide peptidase A (SppA) plays a crucial role in bacterial protein secretion by degrading residual signal peptides after protein translocation, yet its function in *F. columnare* physiology and virulence has not been characterized. Here, we employed a targeted in-frame gene knockout approach to investigate the role of *sppA* in *F. columnare*. The Δ*sppA* mutant exhibited pleiotropic phenotypes including increased outer membrane vesicle (OMV) production (3.8-fold higher compared to the wild type), loss of gliding motility, enhanced efflux pump activity at the transcriptomic level, and reduced virulence. Transcriptomic profiling of the Δ*sppA* mutant revealed significant upregulation of genes involved in membrane stress response, including the genes in the MacAB-TolC efflux system, *algU*, and *osmC*, compared to the wild-type state. Importantly, survival assays demonstrated its virulence was significantly attenuated in freshwater Medaka (*Oryzias latipes*), with a 20% higher survival rate of fish compared to the wild type. Our findings reveal that SppA is essential for maintaining membrane homeostasis and normal cellular physiology in *F. columnare* and serves as one of the virulence factors during columnaris infection. These results provide important insights into the biological function of the *sppA* gene in *F. columnare* and highlight its role in bacterial protein secretion, membrane homeostasis, and pathogenesis.

## Introduction

*Flavobacterium columnare* is a Gram-negative, rod-shaped bacterium that causes columnaris disease in freshwater fish worldwide (1, 2). This disease affects diverse fish species, including catfish, tilapia, and ornamental fish, resulting in substantial economic losses to aquaculture industries (3, 4). The pathogen is characterized by its ability to form biofilms, exhibit gliding motility, and secrete various extracellular proteins through the type IX secretion system (T9SS) (5–7). Despite its economic importance, the molecular mechanisms of *F. columnare* pathogenesis remain poorly understood, and effective control strategies are limited due to the incomplete characterization of virulence factors.

Protein secretion is a key determinant of bacterial pathogenicity (8). The secretion of virulence factors involves the coordinated action of multiple systems. The Sec system serves as the basic transport machinery, responsible for transporting newly synthesized proteins across the inner membrane into the periplasm (9). The T9SS is a secretion system unique to the *Bacteroidota* phylum, responsible for transporting various proteins including virulence factors to the cell surface or to the extracellular environment (10, 11). Although the T9SS selects substrates by recognizing C-terminal domain (CTD) signals, these substrates must first be transported to the periplasm via the Sec system (12). Therefore, the function of the T9SS depends on the normal operation of the Sec system. The continuous flow of proteins through the Sec system generates signal peptide fragments embedded in the membrane, which must be effectively cleared to prevent blockage of the transport channel and maintain secretory capacity.

Signal peptide peptidase A (SppA) is a membrane-bound protease that plays a crucial role in the protein secretion system (13). The proteins to be secreted are initially produced with an N-terminal signal peptide that guides their targets to cross the cytoplasmic membrane. Following protein translocation via the Sec system, signal peptidase cleaves the signal peptide, leaving it embedded in the membrane. SppA then performs intramembrane proteolysis, cleaving within the hydrophobic core of these residual signal peptides (14). SppA is thus responsible for degrading these residual signal peptides and preventing their accumulation, which could otherwise disrupt membrane function and interfere with continued protein translocation (15). Studies in *Bacillus subtilis* have demonstrated that *sppA* deletion leads to signal peptide accumulation, resulting in membrane stress, impaired Sec translocon function, and reduced protein secretion efficiency (16). Given that the T9SS-dependent virulence factors must first traverse the inner membrane via the Sec system, SppA function is potentially critical for maintaining the protein secretion capacity necessary for *F. columnare* pathogenesis.

In a previous study, we developed an attenuated vaccine strain of *F. columnare* through antibiotic-induced mutagenesis and identified missense mutations in the *F. columnare sppA* gene (17), suggesting that *sppA* may be a potential virulence factor contributing to the loss of virulence. In addition, given the essential role of SppA in protein secretion and the dependence of T9SS substrates on Sec-mediated translocation, we hypothesized that SppA is a potential virulence factor for *F. columnare* pathogenesis by ensuring efficient clearance of signal peptides and maintaining membrane homeostasis. Therefore, this study aimed to investigate the biological functions of SppA and evaluate its contribution to bacterial virulence in the bacterial fish pathogen *F. columnare*.

## Materials and methods

### SppA structure analysis

Based on the DNA sequence of the *sppA* gene from *F. columnare* strain HLCZX-1, the corresponding amino acid sequence was subjected to bioinformatics analysis. The physicochemical properties of the SppA protein were predicted using ExPASy ProtParam (https://www.expasy.org/). To determine the phylogeny of the SppA protein, a maximum likelihood (ML) phylogenetic tree was constructed based on the multiple amino acid sequence alignment MUSCLE (v3.7) (18). The SppA protein sequences from eight selected bacterial species: *Flavobacterium oreochromis* (NCBI Ref. #: WP_088398786), *Flavobacterium psychrophilum* (NCBI Ref. #: BHD32194), *Flavobacterium johnsoniae* (NCBI Ref. #: WP_012023794), *Escherichia coli* (NCBI Ref. #: BAA15557), *B. subtilis* (NCBI Ref. #: CAB14931), *Bacillus licheniformis* (WP_003184383), *Enterococcus faecium* (WP_002294607), and *F. columnare* (NCBI Ref. #: XUP20605) were retrieved from the NCBI database. The ML tree was generated using IQ-Tree (v1.6.12) (19) on the CIPRES web server with LG + I + G4 as the best-fit substitution model (20). Branch support was assessed using 1,000 ultrafast bootstrap replicates (21). Furthermore, predicted proteins were searched for domains using the NCBIfam database via Interpro (https://www.ebi.ac.uk/interpro/entry/ncbifam/).

A combined approach was employed to gain structural and functional insights through sequence comparison. Multiple sequence alignment was performed using MAFFT (v7.0) (22). The aligned sequences were subsequently used for secondary structure prediction with EsPript 3.0 (23).

### Bacterial strains, plasmids, and growth conditions

*F. columnare* strain HLCZX-1 isolated from diseased mandarin fish (*Siniperca chuatsi*) was utilized as the wild-type strain for genetic analysis (24). This strain was previously classified as genetic group 1 within the *F. columnare* species (25), and has been identified as *F. columnare* according to the new classification system (26). *F. columnare* strains were cultured in modified Shieh (MS) agar or broth with shaking at 125 rpm at 28 °C for 24–48 h (27). *E. coli* strains DH5α and S17-1 λ *pir*, used for plasmid construction and bacterial conjugation, respectively, were grown at 37 °C and 250 rpm in lysogeny broth (LB) (28). Plasmids in *E. coli* were selected using 100 μg/mL ampicillin, while selection for plasmids in *F. columnare* was achieved with 5 μg/mL tetracycline. In addition, 1 μg/mL of tobramycin was employed to counterselect against *E. coli* for conjugation experiments. The experimental strains and plasmids used in this study are listed in Table 1, and primers are listed in Table 2.

**Table 1:** Bacterial strains and plasmids used in this study.

Strains or plasmids	Description^a^	Source or reference
Strains		
*F. columnare* HLCZX-1	Wild-type strain	(24)
Δ*sppA*	*sppA* deletion of *F. columnare*	This study
C-*sppA*	Δ*sppA* complemented with *sppA* gene	This study
*E. coli* strain DH5α	Strain used for general cloning	Sangon Biotech (Shanghai, China)
*E. coli* strain S17-1 λ *pir*	Strain used for conjugation	(54)
Plasmids		
pMS43	Plasmid carrying *ompA*; *ompA* was amplified with primer *ompA*-for and *ompA*-rev, and cloned into SacI and KpnI sites of pCP23; Ap^r^ (Em^r^, Cf^r^)	(45)
pRE112	Plasmid carrying *sacB*; *sacB* was amplified with primer *sacB*-for and *sacB*-rev, and cloned into SacI and KpnI sites of pCP23 Ap^r^ (Em^r^, Cf^r^)	(55)
pMD-18T	Cloning vector, Ap^r^	Takara Bio (Dalian, China)
pCP23	*E. coli* - *Flavobacterium* shuttle plasmid; Ap^r^ (Tc^r^)	(56)
pMS75	Suicide vector carrying *sacB*, Ap^r^ (Tc^r^)	(29) and this study
pRX01	Recombinant plasmid used to delete *sppA*; 2,457-bp of the upstream region, and 2,559-bp of the downstream region of *sppA* were cloned into the suicide plasmid pMS75; Ap^r^ (Tc^r^)	This study
pCP23-*sppA*	Plasmid for complementation of *sppA* in Δ*sppA*; *sppA* was amplified with primer P23-*sppA*-F/P23-*sppA*-R and cloned between the BamHI and SphI sites of pCP23; Ap^r^ (Tc^r^)	This study

^a^Antibiotic resistance phenotypes: ampicillin (Ap^r^), tetracycline (Tc^r^), erythromycin (Em^r^), and cefoxitin (Cf^r^). Unless indicated otherwise, the antibiotic resistance phenotypes are those expressed in *E. coli*. The antibiotic resistance phenotypes given in parentheses are those expressed in *F. columnare* but not in *E. coli*.

**Table 2:** Sequences of primers used in this research.

Target genes	Primers	Sequence(5′-3′)^a^
*ompA*	*ompA-*for(SacI)	ACGC**GAGCTC**GGCAGCGCATACCAAAGAACA
	*ompA-*rev	TGCAAACTTTTTGAT GTTCATTTTTTAATTACAATTTAGTTAAT
*sacB*	*sacB-*for	ATTAACTAAATTGTAATTAAAAAATGAACATCAAAAAGTTTGCA
	*sacB-*rev(KpnI)	CGG**GGTACC**TTATTTGTTAACTGTTAATTGTCC
*Upstream region of sppA*	*sppA*-L-F(KpnI)	acagttaacaaataa**ggtacc**ATGAAAAAGGCAATACTAGCAGTAGC
	*sppA*-L-R	aaggaagtatGATCATGCAGAATATGAAAAGACCA
	S-LEFT	CAGAACACCGATAGATTACTC
*Downstream region of sppA*	*sppA*-R-F	ctgcatgatcATACTTCCTTATGAGTTAAAAATAAATTAAATG
	*sppA*-R-R (BamHI)	caggtcgactctaga**ggatcc**ATTTACATCACCTCCTAGATATTTAGCTG
	S-RIGHT	TAATAGAGAGGCTCCAATACC
*sppA partial gene 1*	S-L	AGGCACTTTACTAGCTGGCG
	S-R	GAGGTTAGGGCGTTTCCTCC
*sppA with its promoter*	P23-*sppA*-F (BamHI)	cggaccggtacccgg**ggatcc**ATTATATGATCAAGTTTCTCCCCTTGA
	P23-*sppA*-R (SphI)	gattacgccaagctt**gcatgc**GCTTATATCAGCATCAGCAAATGC
*sppA partial gene 2 (qPCR)*	S-q-F	TAGGTTCGGGAGAAGGGGAT
	S-q-R	GCAGCCAGATTGCCCATAGA
*lemA*	*lemA*-F	CGCTCTAAGGCTACACAAGTAACAG
	*lemA*-R	GATAATGCTGACGAAACACCACTTTG
*tolC*	*tolC*-F	AGTAAACGCTGGTGTAGTTCCTAAAG
	*tolC*-R	CACTAACGACCCTTTGAGTATTTGTTG
*macA*	*macA*-F	TCAGGCGAATGTTCAAGTGGATG
	*macA*-R	TGAAGTGGTTAAGTTAGCGGAGTTAG
*macB_1*	*macB*_1-F	GCTTTCTTTGTTAGGCGTTACTATTGG
	*macB*_1-R	CACCGCACTATTATCTAAACTCCCTAC
*tonB_1*	*tonB*_1-F	GACTGCTTCTACTTATGCCGAGTTG
	*tonB*_1-R	AAAGGGATCAAGGGTTGTGTTTCTG

^a^Bold sequences indicate added restriction enzyme sites.

### Construction of pMS75 suicide plasmid for gene editing

Suicide vector pMS75 was re-constructed as previously described (29). In brief, the promoter region of the *Flavobacterium johnsoniae ompA* gene was amplified by PCR using pAS43 as the template, with r-Taq DNA polymerase (Takara) and the primers *ompA*-for (introducing a SacI site) and *ompA*-rev. Similarly, the *sacB* gene was amplified from pRE112 using the primers *sacB-*for and *sacB-*rev (introducing a KpnI site). These two fragments were linked using overlap extension PCR and cloned into the pMD-18T vector (Takara), adding the *ompA* promoter in front of the *sacB* gene to replace its original promoter. The purified PompA + *sacB* fragment, released from the pMD-18T vector after digestion with SacI and KpnI and proliferated in *E. coli* DH5α. The pMD-18T_sacB fragment was excised by the KpnI and BamHI and then ligated into the pCP23 vector, which had been digested with the same enzymes, to generate the suicide plasmid pMS75.

### Conjugative transfer of plasmids in *F. columnare*

Plasmids were transferred from *E. coli* S17-1 λ *pir* to *F. columnare* by conjugation as described previously (30). Briefly, 50 μL of overnight *E. coli* culture was inoculated into 5 mL of LB broth, and 50 μL of overnight *F. columnare* culture was inoculated into 5 mL of MS broth. The cultures were incubated with shaking at 37 °C and 28 °C, respectively, until their optical densities at 600 nm (OD_600_) reached 0.5. The bacterial solution was then centrifuged at 8,000 × *g* for 1 min, followed by washing and resuspending in MS broth. Suspensions of *E. coli* and *F. columnare* were mixed and then centrifuged again at 8,000 × *g* for 1 min to remove excess medium. The cell mixture was resuspended in 200 μL of MS broth, spotted on MS agar, and incubated at 30 °C for 24 h. After incubation, the cells were scraped off the agar and resuspended in 1 mL of MS broth. Then 100-μL aliquots were spread on MS agar containing 1 μg/mL tobramycin and 5 μg/mL tetracycline and incubated at 28 °C for 72 h.

### Construction of *sppA* deletion mutant

In-frame deletion was generated using established protocols (29). Briefly, a 2,457 bp of the upstream fragment of *sppA* was amplified from the *F. columnare* HLCZX-1 genomic DNA using the primer pair *sppA*-L-F (containing the KpnI site) and *sppA*-L-R, while a 2,559 bp downstream fragment was amplified using *sppA*-R-F and *sppA*-R-R (containing the BamHI site) (Table 2). The amplified product was assembled with linearized pMS75 that was digested with KpnI and BamHI using ClonExpress Ultra One Step Cloning Kit V2 (Vazyme), resulting in a recombinant plasmid pRX01. The plasmid pRX01 was introduced into the *F. columnare* HLCZX-1 wild-type strain via conjugation. Colonies with the plasmid integrated into the chromosome were selected based on tetracycline resistance. These colonies were streaked onto MS agar with tetracycline, and isolated colonies were subsequently cultured in liquid MS medium without tetracycline to facilitate plasmid loss by DNA recombination. The cultures were then plated on MS medium containing 10% sucrose, allowing colonies that had lost the *sacB*-containing plasmid to grow, since *sacB* is lethal to cells in the presence of sucrose. Finally, the *sppA* deletion mutant was verified in the colonies growing with sucrose by PCR and whole genome sequencing, and the mutant was designated as Δ*sppA*.

### Complementation of the *sppA* mutant

A 2229 bp fragment spanning the *sppA* gene with its native promoter region was amplified using the primers P23-*sppA*-F (introducing the BamHI site) and P23-*sppA*-R (introducing the SphI site) (Table 2). The amplified product was ligated into the shuttle vector pCP23 to produce a recombinant plasmid pCP23-*sppA*, followed by introduction into the *F. columnare sppA* mutant via conjugation.

### Growth curve analysis

*F. columnare* strains were revived from frozen stock by plating on MS agar and incubating at 28 °C for 36 h. The resulting colonies were used to inoculate 10 mL of MS medium, which was incubated overnight at 28 °C with shaking at 125 rpm. For strains carrying plasmids, 2.5 *μ*g/mL tetracycline was added to the medium. Overnight cultures of the wild type, Δ*sppA* mutant, and the complementary strains were adjusted to an OD₆₀₀ of 0.5 and used to inoculate 3 mL of fresh MS broth. The cultures were incubated at 28 °C with shaking at 125 rpm, and OD₆₀₀ values were measured every 9 h for 54 h using a DEN-600 spectrophotometer (Biosan, Latvia). Growth rates (*μ*) were calculated from the slope of the linear regression line obtained by plotting the natural logarithm of optical density (ln OD_600_) against time during the exponential growth phase. Only time points within the exponential phase were used. Doubling times (tₙ) were then calculated using the formula: t_d_ = ln2/μ.

### Biochemistry and antibiotic resistance test

For biochemical characterization, the oxidase (Oxidase reagent; bioMérieux) and catalase (Oxidase reagent; bioMérieux) analysis, in addition to API 20NE test system (bioMérieux, France) were conducted according to the manufacturer’s instructions in order to identify any biochemical differences between the mutant and wild-type strains. In addition, the antimicrobial susceptibility of *F. columnare* wild-type and Δ*sppA* mutant strains was evaluated against three aquaculture-relevant antibiotics: oxytetracycline dihydrate (OTC), enrofloxacin (ENRO), and florfenicol (FF), which were obtained from Aladdin (Shanghai, China). The broth microdilution method was employed to determine minimum inhibitory concentrations (MICs) following standardized procedures. Overnight bacterial cultures were adjusted to an OD_600_ of 0.04 ± 0.005 in MS broth before testing. Two-fold serial dilutions of each antibiotic were prepared in 96-well microtiter plates (Corning 167,008, Corning, NY), covering a concentration range from 16 μg/mL to 0.00375 μg/mL. Each well received 100 μL of antibiotic solution, followed by inoculation with 20 μL of bacterial suspension, with all concentrations tested in triplicate. Following a 48-h incubation at 28 °C in the dark to prevent antibiotic degradation, MIC values were determined as the lowest antibiotic concentrations that completely inhibited visible bacterial growth.

### Biofilm formation assay

The biofilm formation capability in wild-type, mutant, and complemented strains was assessed using the methodologies described in previous research (31). In brief, cells were grown in MS medium to the mid-logarithmic phase (OD₆₀₀ = 0.5). The cultures were diluted 1:100 in MS medium, and 150 μL of the diluted culture was added to each well of a 96-well flat-bottom polystyrene microplate (Corning 167,008, Corning, NY). The plate was covered with aluminum foil and incubated at 28 °C for 24 h. Biofilm formation was tested in three wells per strain, with sterile, uninoculated medium serving as a negative control. After incubation, the medium was discarded, and the wells were washed three times with 200 μL of sterile distilled water. Each well was then stained with 150 μL of 1% (w/v) crystal violet solution for 30 min at room temperature. Excess dye was removed by washing the wells four times with 200 μL of sterile distilled water. The bound crystal violet was dissolved with 100 μL of ethanol, and absorbance was measured at 595 nm (OD₅₉₅) using a SpectraMax iD3 Multi-Mode Microplate Reader (Molecular Devices, USA). The absorbance of the uninoculated negative control was subtracted from the absorbance of each strain.

### Analysis of cell motility and colony morphology

Gliding of individual cells was observed microscopically after cells were grown with shaking at 28 °C in 1/10 MS broth overnight with shaking; tunnel slides were prepared using double-sided tape, glass microscope slides, and coverslips as previously described (32), then 10 μL of culture was added to each tunnel, incubated for 5 min, and cell motility was recorded at 25 °C using a Nikon Ci-L plus microscope equipped with an SC2000C CMOS camera, with rainbow traces of cell movement generated using Fiji version 2.14.0 (https://imagej.net/software/fiji/) and the Color FootPrint macro (33). Colony morphology was examined by serial dilution plating. In brief, mid-exponential phase cultures were serially diluted (10-fold dilutions) in sterile MS medium, and 100 μL of the appropriate dilutions were spread onto MS agar plates. After incubation at 28 °C for 48 h, individual colony morphology was observed and photographed using a Nikon SMZ25 microscope (Tokyo, Japan).

### Transmission electron microscopy (TEM) analysis

For TEM analysis, bacterial cells from triplicate overnight cultures were harvested by centrifugation and immediately fixed in TEM-grade fixative at 4 °C. The fixed cells were washed three times with 0.1 M phosphate buffer (PB, pH 7.4) for 3 min per wash, resuspended in 1% agarose solution, and encapsulated before the agarose solidified. The agarose blocks were post-fixed with 1% osmium tetroxide (OsO₄) in 0.1 M PB (pH 7.4) for 2 h at room temperature in the dark, followed by three rinses in 0.1 M PB. The samples were dehydrated at room temperature through a graded ethanol series (30, 50, 70, 80, 95, and 100% ethanol) and two changes of acetone. Resin infiltration was performed with acetone and EMBed 812 resin mixtures (1:1 for 2–4 h, 1:2 overnight, and pure resin for 5–8 h) at 37 °C, after which the samples were embedded in pure resin and cured overnight at 37 °C. Polymerization was carried out at 60 °C for over 48 h. Ultrathin sections (60–80 nm) were prepared using an ultramicrotome, then placed on 150-mesh copper grids coated with formvar film and stained with 2% uranyl acetate and 2.6% lead citrate. The stained sections were observed under TEM, and detailed images were captured.

For OMV quantification, 10 micrographs were randomly selected from each of the three independent biological replicates per strain (WT and Δ*sppA*), yielding a total of 30 micrographs per strain. To prevent observer bias, OMV counting was conducted in a blinded manner, with images coded prior to analysis. OMVs were defined as spherical, membrane-bound vesicular structures with diameters ranging from 20 to 250 nm, observed either as active outer-membrane budding events or as free vesicles in the immediate pericellular region (34). For accurate quantification and normalization, the total number of OMVs was counted and divided by the number of clearly identifiable, intact bacterial cells present in each micrograph. The final quantitative data are expressed as the average number of OMVs per cell.

### Fish challenge

Wild-type *F. columnare* and Δ*sppA* mutant strains were cultured overnight in MS medium for 24 h at 28 °C. On the following day, 50 μL of these cultures were transferred into 5 mL of fresh MS broth and incubated with shaking at 28 °C until the OD_600_ reached 0.4. To quantify viable cells, serial dilutions (in triplicate) of the cultures were plated and enumerated on MS agar. Freshwater medaka fish were hatched and obtained from the fish husbandry unit of the State Key Lab of Marine Pollution (City University of Hong Kong) and were used for bacterial challenge. No signs of disease were observed before the challenge, and no evidence of *F. columnare* infection was detected in the uninfected control tanks and acclimation tanks throughout the study. Fish were transferred to challenge aquaria one week prior to the immersion challenge for acclimation.

Challenges were performed using triplicate 1-liter beakers with restricted water flow at 28 °C, each containing 10 fish. For the immersion challenge, water flow was stopped, and bacterial cultures were added to the beakers, which were then incubated for 2 h before resuming water flow. Control beakers were inoculated with MS broth instead of bacterial cultures. The final challenge concentrations for the experiment were 7 × 10^5^ CFU/mL for the wild-type *F. columnare* and 1.05 × 10^6^ CFU/mL for the Δ*sppA* mutant. Mortalities were monitored, removed, and recorded daily. Data from the triplicate beakers for each strain were combined, and survivor fractions were calculated. The deceased fish were randomly selected and subjected to bacterial examination to confirm the columnaris infection. Swabs from external and internal organs were streaked on MS agar to identify the plausible yellow, rhizoid, and adherent colonies of *F. columnare*. The present experiment was conducted in compliance with the animal research guidelines of the Hong Kong Special Administrative Region (HKSAR) under animal license [Ref No.: (24–50) in DH/HT&A/8/2/5 Pt.14] and with approval from the City University Animal Ethics Committee (Approval No.: A-0402).

### RNA isolation and transcriptomic analysis

Wild-type *F. columnare* and the Δ*sppA* mutant were cultured in MS broth until they reached early stationary phase (OD_600_ = 0.8). Total RNA was extracted using the RNeasy Mini Kit (QIAGEN, Valencia, CA) and subsequently treated with DNase I to eliminate any contaminating DNA. The quality and quantity of RNA were assessed using NanoDrop and Agilent 2,100 instruments (Agilent Technologies, Palo Alto, CA). Three biological replicates were conducted for each strain. Ribosomal RNA depletion and library construction were conducted by the Novogene company (Beijing, China). Quantified libraries were pooled and sequenced on Illumina NovaSeq X Plus to generate the raw data of each sample. The qualities of the resulting raw reads were first evaluated by the FastQC program (v0.11.8) (35). Low-quality sequences and adapters were trimmed using Trimmomatic v0.32 (36). High-quality RNA-seq sequences were then mapped to the *F. columnare* reference genome (GCF_050711845.1) using Bowtie2 (37). Gene expression levels were quantified using FeatureCounts software (v2.0.3) (38). Differentially expressed genes (DEGs) between the wild-type and Δ*sppA* strains were identified using DESeq2 (version 1.34.0) (39). Significantly differentially expressed genes were considered by parameters: |log2(fold change)| > 1 and false discovery rate (FDR) < 0.05 (27). Raw sequencing reads were deposited in the NCBI Sequence Read Archive (SRA) as part of BioProject PRJNA1321558.

### Quantitative real-time PCR validation

To further validate the RNA-seq data, five representative DEGs were selected for expression quantification using quantitative real-time PCR (qPCR) on an Applied Biosystems QuantStudio 7 Pro system (Thermo Fisher) with gene-specific primers (Table 2). In brief, total RNA was extracted using the RNeasy Mini Kit (QIAGEN) and quantified by NanoDrop 2000 spectrophotometry (Thermo Fisher). cDNA synthesis was performed using HiScript IV All-in-One Ultra RT SuperMix (Vazyme) following the manufacturer’s protocol. qPCR reactions were carried out with Taq Pro Universal SYBR qPCR Master Mix (Vazyme) under the following cycling conditions: initial denaturation at 95 °C for 30 s, followed by 40 cycles of 95 °C for 10 s and 60 °C for 30 s, with a final melt curve analysis of 95 °C for 15 s, 60 °C for 60 s and 95 °C for 15 s. The gene *gap1* was used as an internal control, and relative gene expression was calculated using the 2^−ΔΔCT^ method (31).

### Statistical analyses

All experiments were conducted with at least three biological replicates. Data were presented as mean ± standard deviation. Statistical analyses were performed using SPSS 16.0. Significance was determined by Student’s t-test or one-way ANOVA followed by LSD test. *p < 0.05* was considered statistically significant. GraphPad Prism version 10 (GraphPad Software, LLC) was also used to compute statistical tests.

## Results

### Identification of the *sppA* gene in the *F. columnare* genome

In a previous study, missense mutations were identified in the *sppA* gene in a rifampicin-resistant attenuated mutant of *F. columnare.* Using this sequence, we performed a BLAST search against the genome of strain HLCZX-1 and identified a single-copy gene (locus tag: V8245_05745, RefSeq: WP_077225657.1) located at 1,361,425-1,363,194 on the complementary strand. Domain analysis confirmed typical SppA features, and the gene was selected for functional characterization. Further sequence analysis revealed that this gene contains an open reading frame of 1,770 bp, encoding a polypeptide of 589 amino acids. The theoretical molecular weight of the protein was calculated to be 66.09 kDa, with a predicted isoelectric point (pI) of 5.57.

**Figure 1:**
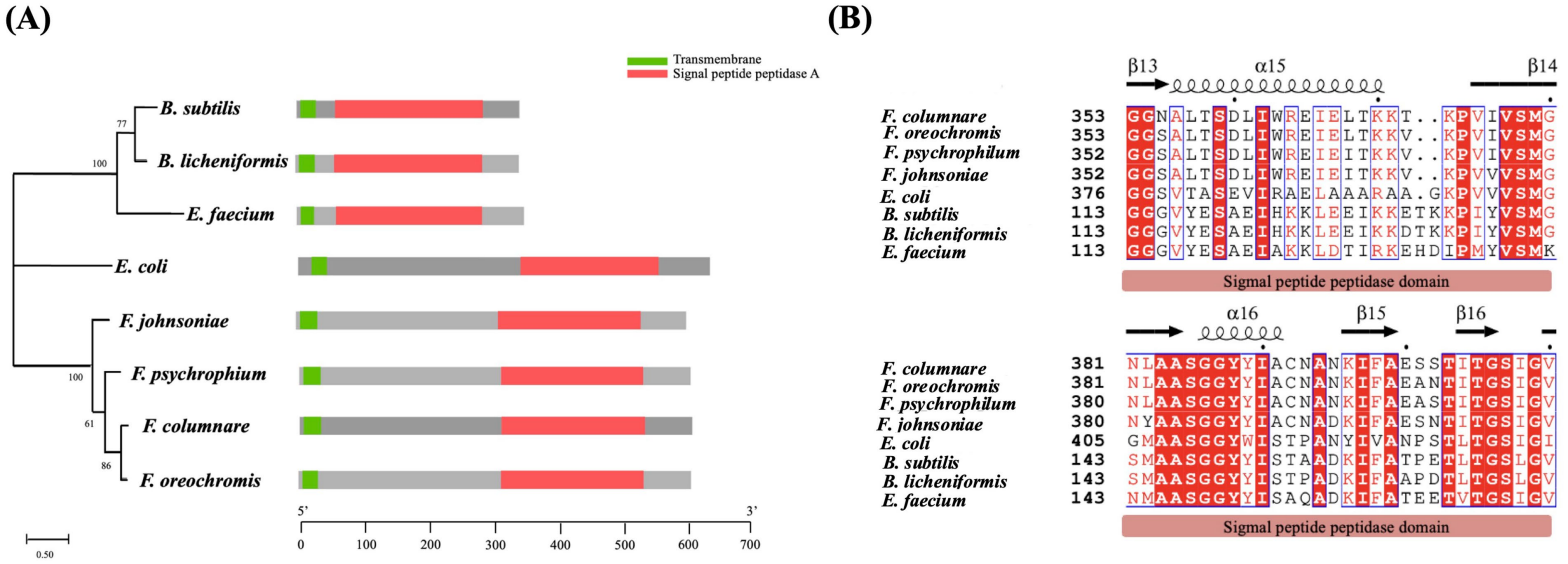
Comparative analysis of SppA across representative bacterial species. **(A)** Phylogeny and domain architecture of SppA Proteins. The SppA protein sequences from 8 different bacterial species were used to construct an ML tree with 1,000 bootstrap replications using the CIPRES software. Lengths of each domain are displayed proportionally. The green boxes represent transmembranes, and the red boxes represent signal peptide peptidase domains. **(B)** Partial multiple sequence alignment and secondary-structure annotation of SppA homologs. Alignments were performed using MAFFT and Espript 3.2. Identical residues are represented on a red background, and residues that are conserved across groups are boxed in blue. The secondary structures of SppA are depicted on top.

Proteins with shared domains often exhibit evolutionary and functional relatedness. To study the conservation of SppA among bacteria, the sequences of SppA were compared with those of other well-studied bacteria. Since the function of SppA was well-studied in *E. coli* and *B. subtilis*, we conducted pairwise alignments of the SppA of these three strains together with other representative bacteria. Sequence comparison revealed that *F. columnare* SppA shares 32.7% amino acid sequence identity with *E. coli* SppA and 32.0% with *B. subtilis* SppA, and they share the two key functional domains, including a short N-terminal transmembrane helix and a signal peptide peptidase domain (green/red in Figure 1A). The results showed a high level of conservation of the signal peptide peptidase domain, indicating functional conservation of SppA within bacterial evolution (Figure 1B; Supplementary Figure S1).

### Generation of the Δ*sppA* and the complemented strain

**Figure 2:**
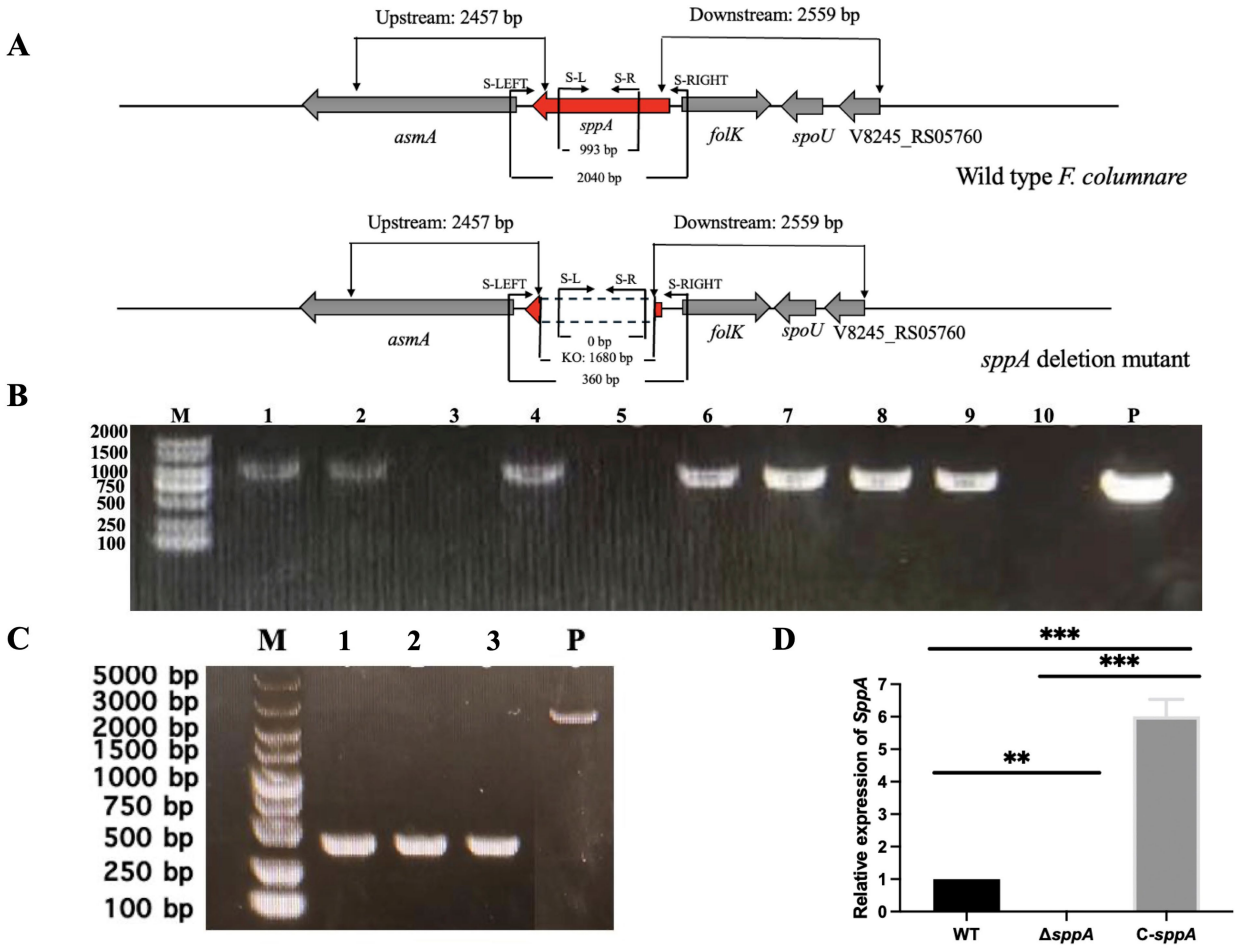
Construction of the *sppA* mutant and the complemented strain. **(A)** Genomic map of *F. columnare sppA* and its deletion. The numbers shown above and below the map indicate the length of the amplified sequence. Primer binding sites used to generate deletion or complementary constructs in PCR reactions are shown above the map. Deleted regions in the mutant are indicated by dashed lines. **(B)** PCR screening of the mutant strains (Δ*sppA*). The primers S-L and S-R, which were designed within the deleted region of *sppA*, did not amplify a product from the Δ*sppA*, while they amplified the corresponding bands (993 bp) from the wild-type. Lanes 1, 2, 4, 6, 7, 8, and 9 correspond to colonies that produced the expected wild-type band, indicating the presence of the wild-type allele. Lanes 3, 5, and 10 did not yield a PCR product, which is consistent with the Δ*sppA* deletion genotype, but may also result from PCR failure; therefore, additional confirmation (see panel **C**) was performed. Lane “P” contains wild-type genomic DNA as the PCR template, serving as a wild-type control. **(C)** PCR confirmation of *sppA* gene deletion mutants. The forward primer S-LEFT and reverse primer S-RIGHT, designed within the upstream and downstream sequences of the deleted region, were used in the PCR. The bands amplified in the deletion mutants were significantly smaller than those obtained in the wild-type strain, with the difference being the number of DNA bases in the deleted region (1,680 bp). Lanes 1, 2, 3 correspond to three independent Δ*sppA* mutants identified in the PCR screening shown in panel B (i.e., lanes 3, 5, 10), while lane “P” contains wild-type genomic DNA as the PCR template. The two PCR tests indicated that these strains were the correct *sppA* gene deletion mutants (Δ*sppA*). **(D)** Detection of the *sppA* expression. The expression levels of the *sppA* gene in the wild-type *F. columnare*, ∆*sppA*, and C-*sppA*, were determined by qPCR. The data are presented as mean ± standard deviation (SD) from three independent biological replicates. ** *p* < 0.01, *** *p* < 0.001.

In order to generate the Δ*sppA*, the suicide plasmid pMS75 was successfully constructed and used to create the intragenic deletion mutant Δ*sppA* through double-crossover homologous recombination. Whole genome sequencing confirmed the precise deletion of the *sppA* gene and revealed no secondary or off-target mutations elsewhere in the genome (data not shown). Furthermore, the full-length *sppA* gene, along with its native promoter region, was cloned into the shuttle vector pCP23 and introduced into the Δ*sppA* mutant via conjugation, resulting in the complementation strain C-*sppA*. The successful construction of the Δ*sppA* and the complementation strain was verified using PCR analysis (Figure 2).

### Growth dynamics and biochemistry test

**Figure 3:**
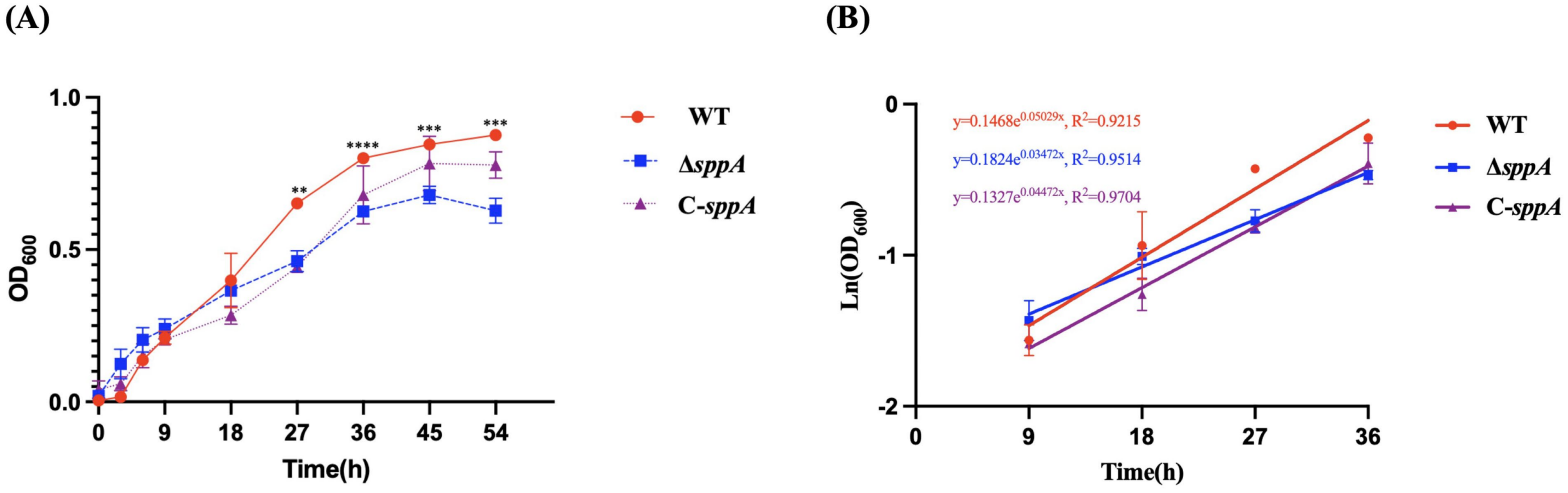
Growth curves **(A)** and growth rate estimates **(B)** of wild-type *F. columnare* (WT, red), Δ*sppA* mutant (Δ*sppA,* blue), and complemented strain (C-*sppA,* purple), respectively. The bacteria were cultured in MS broth at 28 °C with shaking (125 rpm), and OD_600_ was measured every 9 h for 54 h. Growth curves were performed in triplicate, and error bars are presented as means ± SD. Statistical difference was assessed using a two-tailed Student’s t-test. ^**^ *p <* 0.01, ^***^ *p <* 0.001, or ^****^ *p <* 0.0001 represents a highly significant difference, extremely significant difference, or most extremely significant difference between the wild-type *F. columnare* and the Δ*sppA* mutant.

Wild-type *F. columnare* and the *sppA* mutant were grown in MS medium, and biomass accumulation was tracked as OD_600_ (Figure 3A). The strains showed comparable lag phases (*p* > 0.05). Thereafter, the curves diverged: the wild type reached significantly higher OD_600_ than Δ*sppA* at 27 and 36 h (log phase) and at 45 and 54 h (stationary phase), with C-*sppA* partially restoring the wild-type phenotype. The Δ*sppA* mutant ceased growth and entered the stationary phase at a significantly lower cell density than the wild-type strain. The growth impairment was partially rescued in the C-*sppA* strain, which exhibited an intermediate phenotype. The growth rates of *F. columnare* strains were determined from 4 time points within the logarithmic phase (Figure 3B). The wild-type strain grew at a rate of 0.050 h^−1^ (doubling time = 13.9 h), whereas the Δ*sppA* mutant exhibited a slower rate of 0.035 h^−1^ (doubling time = 19.8 h), with a statistically significant difference (*p* = 0.009).

Biochemical tests indicated that no key phenotypic differences, such as carbohydrate metabolism and glucose utilization, were detected between the strains using the standardized API test system (data not shown). We investigated the effect of the *sppA* deletion on bacterial antibiotic resistance, and the results showed that the deletion of *sppA* did not alter bacterial resistance to commonly used antibiotics in aquaculture (i.e., OTC, ENRO, and FF) (Supplementary Table S1).

### Effect of deletion of the *sppA* gene on gliding motility and colony spreading

**Figure 4:**
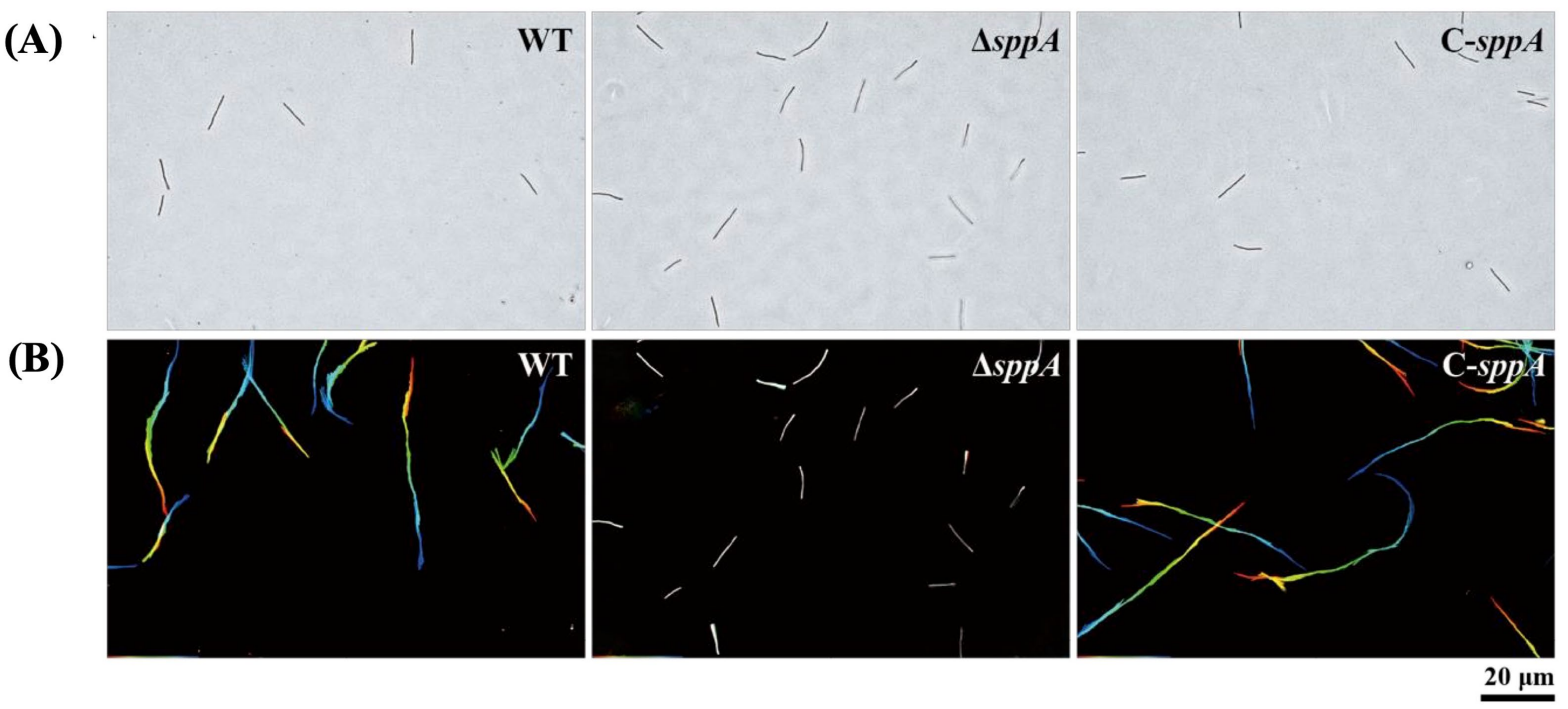
Gliding motility of wild-type *F. columnare*, Δs*ppA* mutant, and complemented strain on glass surfaces. Wild-type *F. columnare*, the Δ*sppA* mutant, and the Δ*sppA* strain carrying wild-type *sppA* on pCP23 (C-*sppA*) were cultured in 1/10 MS medium at 28 °C overnight with shaking; 10 μL of each culture was added to glass tunnel slides, and cell motility was observed using a Nikon Ci-L plus microscope, with single-frame images colored from red (time zero) through orange, yellow, green, cyan, to blue (30 s) and integrated to display the ‘rainbow trace’ of gliding cells. **(A)** The initial frame of the motility video for each strain. **(B)** The corresponding 30-s rainbow trajectories, where white cells indicate minimal movement, the 20 μm scale bar applies to all images, and the rainbow tracks correspond to sequences shown in the Supplementary vedio S1.

**Figure 5:**
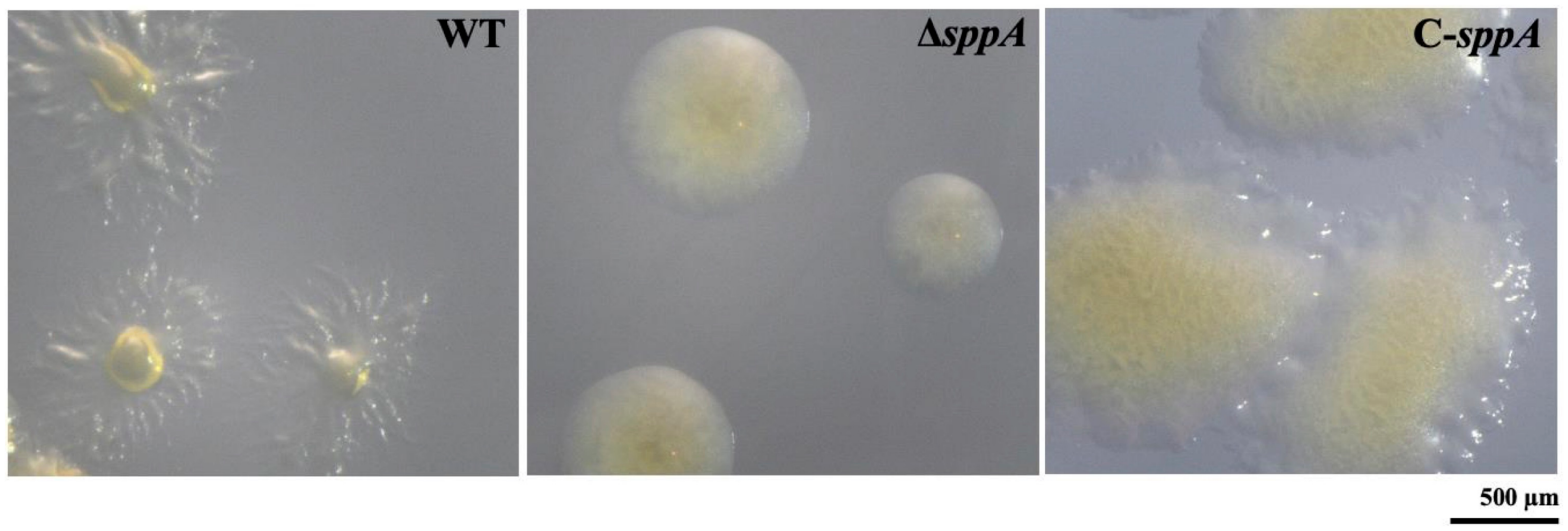
Photomicrographs of wild-type, mutant, and complemented *F. columnare* strains on MS agar. Colonies were cultured for 48 h at 28 °C on MS agar. Strains examined were wild type (WT), Δ*sppA,* and C-*sppA*. Photomicrographs were taken with a Nikon SMZ25 microscope. Images for WT and C-*sppA* strains were characterized for spreading colonies on MS agar, whereas the image of the Δ*sppA* mutant formed non-spreading colonies.

Wild-type cells glide across glass surfaces, whereas the Δ*sppA* mutant displays defective gliding motility on glass, and complementation of the Δ*sppA* mutant restores this motility (Figure 4; Supplementary Vedio S1). In addition, the cells of the wild-type and complemented strains developed thin, spreading colonies on MS agar, whereas the cells of the Δ*sppA* mutant produced non-spreading colonies (Figure 5), possibly due to differences in gliding movements.

### The Δ*sppA* mutant exhibits defects in biofilm formation

**Figure 6:**
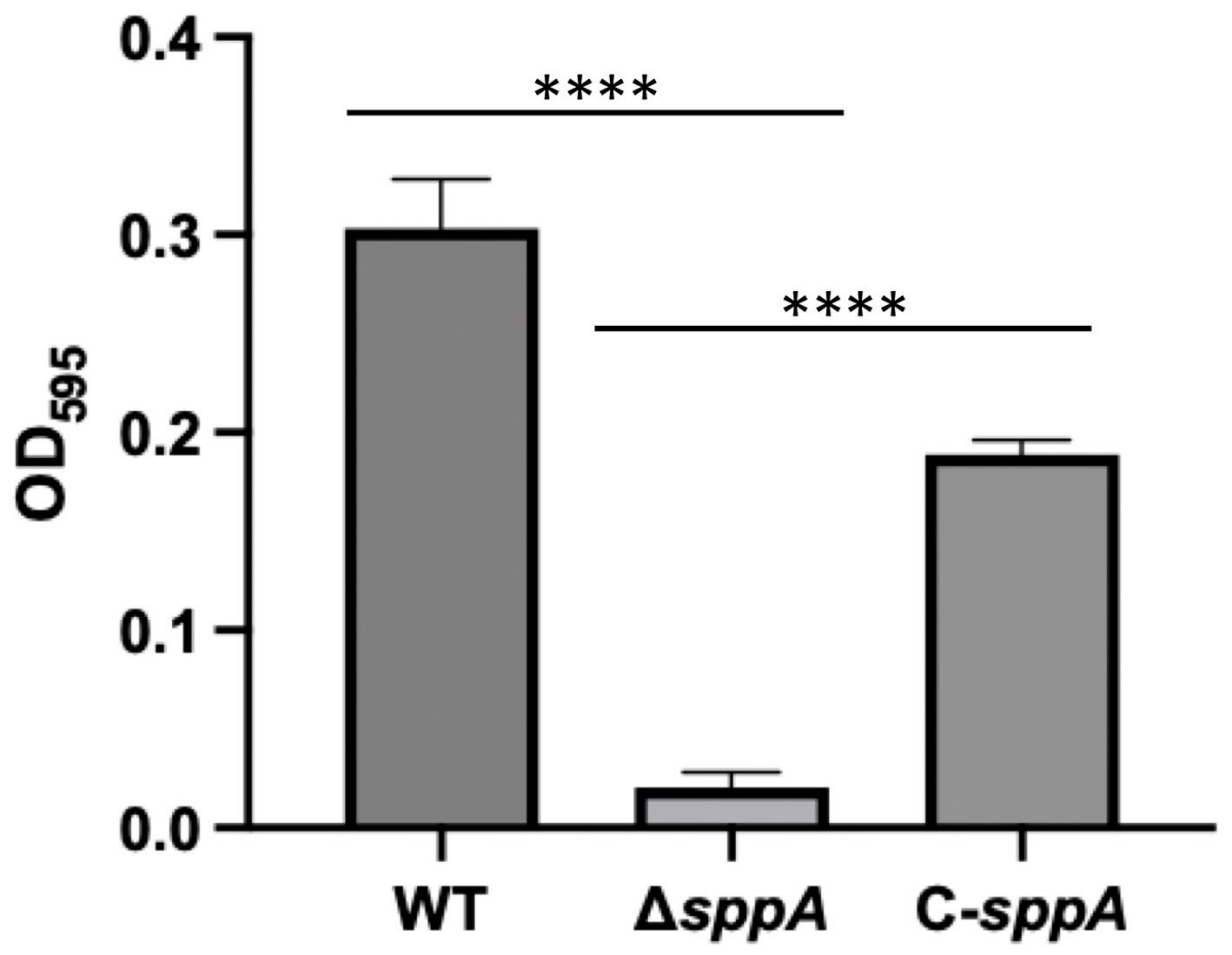
Biofilm formation of wild-type *F. columnare* (WT), Δ*sppA* mutant, and Δ*sppA* mutant complemented with pCP23-*sppA*. Biofilm formation on polystyrene of cells in MS broth incubated for 24 h at 28 °C without shaking. The results are presented as mean ± SD, with each condition tested in triplicate (*n* = 3). Significant differences between groups were determined using one-way ANOVA, where ^****^p < 0.001 indicates levels of statistical significance.

Given that gliding motility facilitates initial surface contact and colonization, we next investigated whether the impaired motility of the *sppA* mutant would affect biofilm development. Crystal violet assays revealed a significant reduction in biofilm biomass in the mutant compared to the wild type, while the complemented strain showed intermediate biofilm biomass between the mutant and wild-type levels (Figure 6).

### Enhanced outer membrane vesicles (OMVs) production in the Δ*sppA* mutant

**Figure 7:**
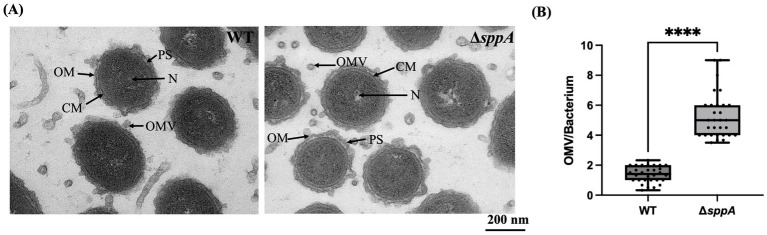
TEM micrographs of wild-type *F. columnare* and the Δ*sppA*. **(A)** TEM observations of wild-type *F. columnare* and the *sppA* gene deletion mutant. Arrows indicate nucleoid (N), cell membrane (CM), outer membrane (OM), outer membrane vesicle (OMV), and periplasmic space (PS). The scale bar represents 200 nm and applies to both images. **(B)** Comparative analysis of OMVs production per cell between wild-type strain and Δ*sppA* mutant strains of *F. columnare*. Data were calculated based on 30 randomly selected images per strain across three independent biological replicates (*n* = 3) and are presented as means ± SD. Statistical significance was determined using Student’s t-test (^****^p < 0.0001).

To assess whether the defect of *sppA* affects cell envelope integrity and protein secretion, TEM was used to analyze the cellular ultrastructure. Interestingly, significant ultrastructural differences were observed between the Δ*sppA* mutant strain and the wild-type strain. The mutant strain showed a significant increase in the number of OMVs around the cells and enhanced definition of the inner and outer membranes (Figure 7A). Quantitative analysis further confirmed that the observed number of OMVs per cell of the Δ*sppA* mutant strain (5.261 ± 1.512) was significantly increased by about 3.8-fold compared with that of the wild type (1.383 ± 0.5439) (^****^p < 0.0001) (Figure 7B). These results suggest that deletion of the *sppA* gene may lead to the accumulation of intracellular proteins, which the cell compensates for by increasing OMV production to extrude misfolded or excess proteins, thereby remodeling the cell envelope structure.

### Virulence of the Δ*sppA* mutant was reduced in the freshwater Medaka infection model

**Figure 8:**
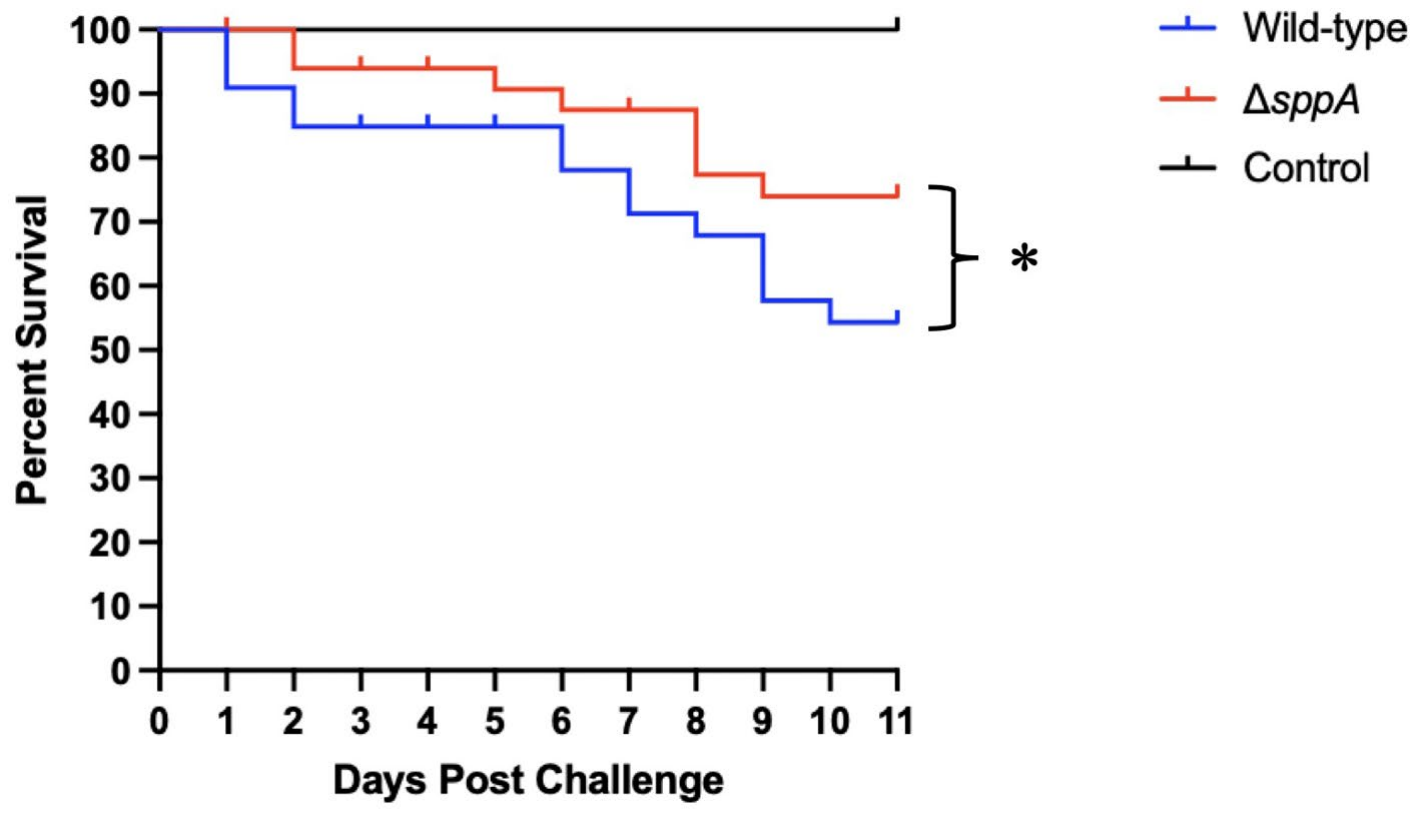
Virulence assessment of *F. columnare* wild type and Δ*sppA* mutant in Medaka fish. Three groups of 30 Medaka fish (*n* = 90) were subjected to exposure to the wild type (indicated in blue) and the Δ*sppA* mutant (shown in red). A control group was exposed to an equivalent volume of MS growth medium (shown in black). Survival data were analyzed using Kaplan–Meier log rank survival analysis, and the significant differences in percent survival for fish challenged were observed between the wild type and Δ*sppA* (*p <* 0.05).

To evaluate the role of *sppA* in pathogenesis, we compared the virulence of wild-type *F. columnare* and the Δ*sppA* mutant in freshwater Medaka fish (Figure 8). The artificial infection experiment revealed that the survival rate in the Δ*sppA* mutant challenge group reached 74.0%, compared to only 54.3% in the wild-type strain treatment group, indicating a significantly higher survival rate in the *sppA* gene knockout group. Fish infected with the Δ*sppA* mutant developed similar columnaris disease symptoms with the wild type counterpart, including yellow external lesions and lethargic swimming, during the 11-day observation period. *F. columnare* colonies were recovered from various internal organs of the deceased fish, confirming the columnaris infection.

### Transcriptomic analysis

**Figure 9:**
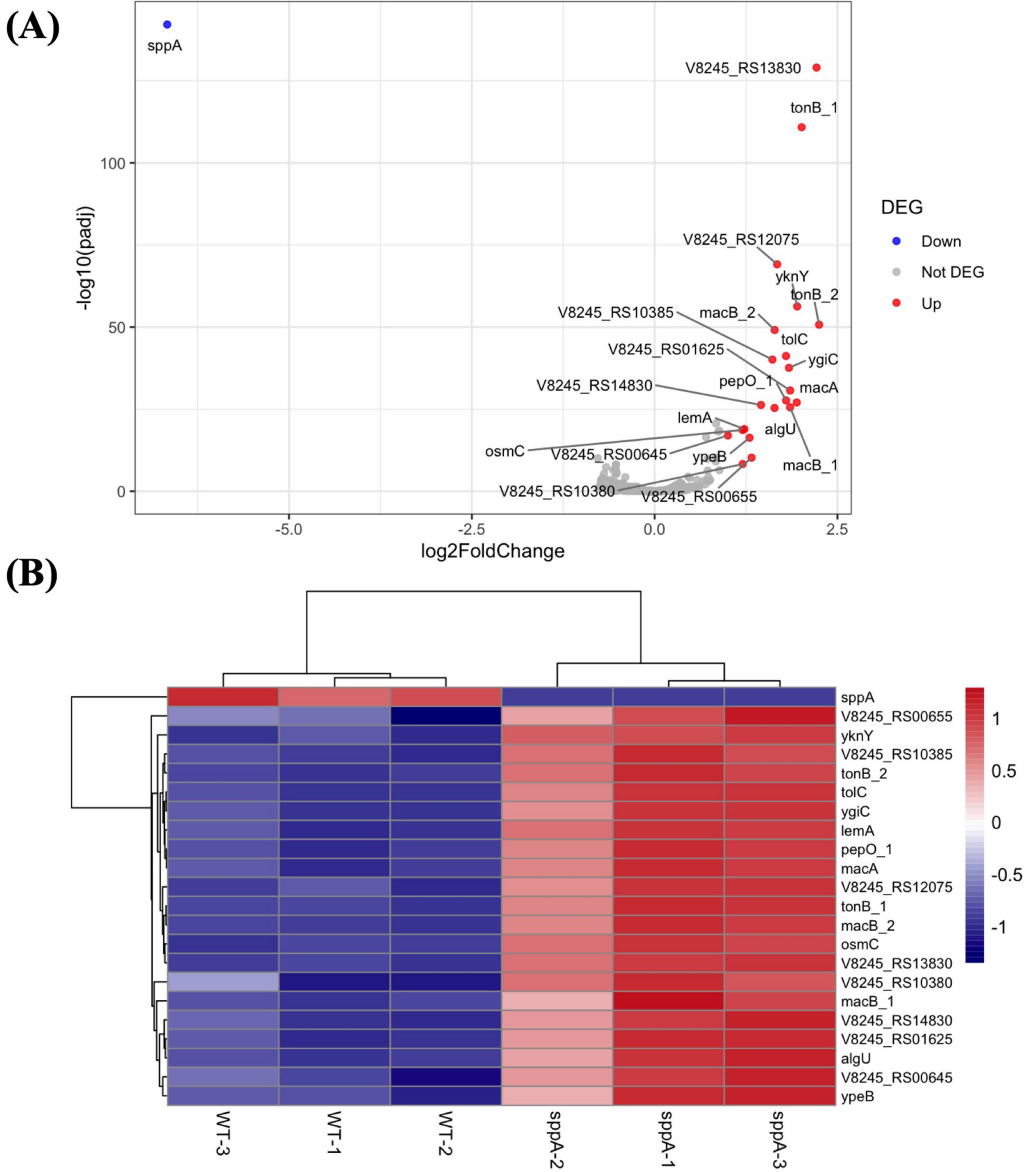
Differentially expressed genes between wild-type *F. columnare* and Δ*sppA*. **(A)** Volcano plot of DEGs comparing Δ*sppA* vs. the WT, p_adj < 0.05 and |log2FoldChange| > 1. The x-axis represents the log2FoldChange, while the y-axis represents the statistical significance of each gene. **(B)** Heatmap of 22 DEGs between wild-type and Δ*sppA* groups. Each row represents a gene, and each column represents a sample, including control (WT-1, WT-2, WT-3) and treatment (*sppA*-1, *sppA*-2, *sppA*-3) groups. Gene expression levels were standardized using z-score normalization, with red indicating high expression and blue indicating low expression.

RNA-seq analysis revealed limited but specific changes in the Δ*sppA* mutant, with only 22 DEGs identified between the wild-type and the Δ*sppA* mutant. As expected, *sppA* (the deleted locus) was the sole downregulated gene, while the remaining 21 DEGs were upregulated (Table 3; Figure 9). The top upregulated genes were associated with stress response, cell membrane maintenance, and efflux pump activities. Notably, genes encoding the MacA/MacB/TolC tripartite efflux pump (e.g., *macA*, *macB*, *tolC*) were significantly upregulated in the Δ*sppA* mutant. This RND-family transporter system is a complex protein secretion system found in bacteria that helps expel toxic compounds out of the cell. Since SppA functions as a protease involved in protein processing and degradation, its absence may lead to the accumulation of misfolded proteins or toxic metabolites, and the MacA/MacB/TolC tripartite efflux pump likely serves as a compensatory mechanism for enhanced efflux pump activity to manage cellular stress resulting from the *sppA* deficiency.

**Table 3:** Representative genes differentially expressed in the *sppA* mutant compared with the wild-type strain.

Gene symbol	Function description		log2FC (Δ*sppA/*WT)
Resistance			
*algU*	RNA polymerase ECF-type sigma factor		1.64
*V8245_RS12075*	Beta-carotene 15,15′-monooxygenase		1.68
*osmC*	Putative stress-induced protein OsmC		1.20
*ypeB*	DUF3820 domain-containing protein		1.30
*yknY*	ABC-type antimicrobial peptide transport system, ATPase component		1.95
*V8245_RS13830*	TPM domain-containing protein		2.21
Cell membrane			
*tolC*	Outer membrane efflux protein		1.80
*macA*	ABC transporter, RND-adapter-like protein		1.94
*macB_1*	ABC-type antimicrobial peptide transport system, permease component		1.85
*macB_2*	Macrolide export ATP-binding/permease protein MacB		1.64
*V8245_RS00645*	Peptidase, M16 family protein		1.00
*lemA*	LemA family protein		1.22
*V8245_RS10380*	Lipoprotein		1.20
*V8245_RS10385*	Lipoprotein		1.61
*ygiC*	Putative acid--amine ligase YgiC		1.84
*tonB_1*	Putative TonB-dependent receptor		2.01
*tonB_2*	Putative TonB-dependent receptor		2.25
*V8245_RS14830*	Glycosyl transferase, group 2 family		1.45
*V8245_RS01625*	Cell shape-determining protein		1.85
Protein processing and degradation			
*pepO_1*	Neutral endopeptidase		1.80
Others			
*V8245_RS00655*	Hypothetical protein		1.32

The upregulation of *algU*, encoding an extracytoplasmic function sigma factor, provides further evidence of stress response activation in the Δ*sppA* mutant. In many bacteria, AlgU-type sigma factors (*algU*) are known to stimulate OMV production and facilitate the removal of periplasmic proteins (40). This finding correlates with our TEM observations showing increased OMV formation in the Δ*sppA* mutant, suggesting that the OMVs may serve as an adaptive response to manage the cellular stress caused by *sppA* deficiency. Additionally, RNA-seq data revealed enhanced expression of genes involved in membrane homeostasis (*tolC*, *macA*, *macB*, *macB_2*, *lemA*, *tonB*), protein processing and degradation (*pepO_1*), and oxidative stress response (*algU*, *osmC*, *ypeB*, *yknY*, TPM domain-containing gene), indicating that *sppA* plays a broader role in maintaining cellular integrity via maintaining its proteolytic function.

**Figure 10:**
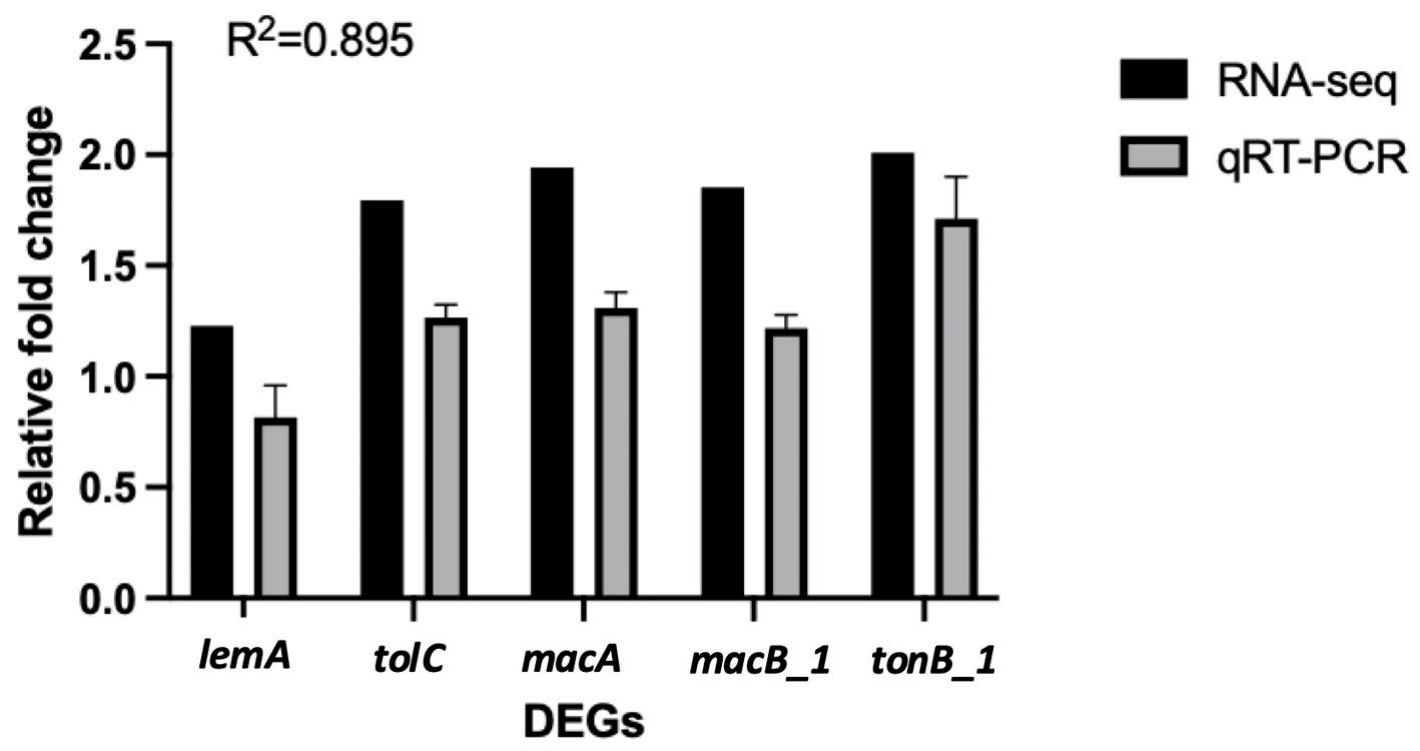
Verification of RNA-seq data by qPCR. Comparison of relative fold change between RNA-seq and qPCR results. Error bars represent the standard error of three biological replicates. Proteins encoded by the analyzed genes: LemA family protein, *lemA*; outer membrane efflux protein, *tolC*; ABC transporter/RND-adapter-like protein, *macA*; ABC-type antimicrobial peptide transport system, permease component, *macB_1*; Putative TonB-dependent receptor, *tonB_1*.

To further validate the transcriptome analysis results, a subset of 5 representative genes selected from the list of DEGs was selected for qPCR validation. Fold changes from qPCR were compared with the RNA-seq expression analysis results. As shown in Figure 10, the qPCR results were significantly correlated with the RNA-Seq data (R^2^ = 0.895), supporting the reliability and accuracy of the transcriptome analysis (Figure 10).

### Proposed compensation mechanism for *sppA* deficiency

**Figure 11:**
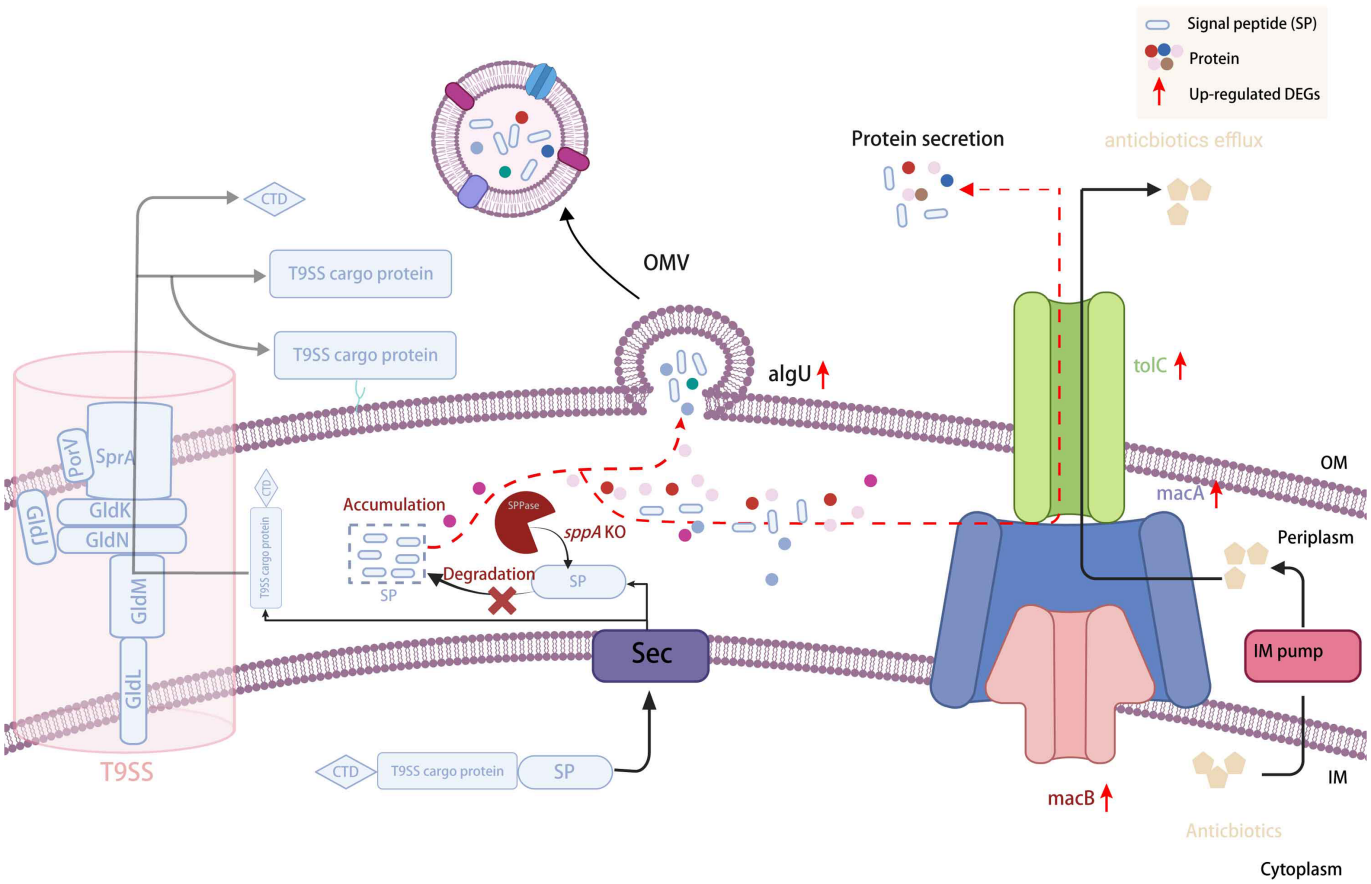
Proposed hypothetical diagram for protein secretion and metabolites efflux in the periplasmic space of the Δ*sppA* mutant. IM, inner membrane; OM, outer membrane; SP, signal peptide; SPPase, signal peptide peptidase; OMV, outer membrane vesicle; T9SS, type IX secretion system.

Based on the experimental findings and the illustrated model, we hypothesize that the deletion of *sppA* in *F. columnare* triggers a cascade of cellular responses to counteract signal peptide accumulation. Following *sppA* knockout, signal peptides that would normally be degraded accumulate in the inner membrane, creating membrane stress and potentially affecting protein translocation efficiency. This accumulation appears to induce compensatory mechanisms, as evidenced by the significant upregulation of the MacAB-TolC efflux pump system, which may serve as an alternative route for removing accumulated peptides or toxic compounds resulting from membrane stress. The increased production of OMVs, confirmed by TEM observations, likely represents another stress response mechanism to alleviate membrane perturbation and potentially export misfolded or aggregated proteins. The activation of the *algU* stress response pathway further suggested that *sppA* deletion creates significant cellular stress. Collectively, these findings demonstrate that *sppA* plays a central role in maintaining cellular homeostasis, and its absence forces the cell to activate multiple stress response pathways, particularly enhanced efflux pump activity and OMV production, to cope with membrane stress (Figure 11).

## Discussion

*F. columnare* represents a major bacterial pathogen affecting commercially important fish species, such as farmed catfish and tilapia, with substantial economic impacts on global freshwater aquaculture (27, 41–44). However, due to the lack of efficient genetic manipulation methods, its virulence factors have been largely unexplored (45, 46). In this study, we successfully constructed a mutant strain (Δ*sppA*) with stable genetic characteristics using in-frame deletion of the gene *sppA* (encoding signal peptide peptidase A) by homologous recombination. This genetic construct enabled a systematic investigation of the gene’s role in bacterial pathogenesis and physiological functions. Using multiple analytical approaches, including ultrastructural characterization, growth kinetics, biofilm quantification, and global transcript profiling, our results revealed a pleiotropic regulatory role of *sppA* in *F. columnare*, with the mutant leading to significant physiological alterations, such as enhanced outer membrane vesiculation, loss of motility, enhanced efflux pump activity at the transcriptomic level, and reduced virulence.

Transcriptome analysis showed that deletion of the *sppA* gene in *F. columnare* significantly altered the expression pattern of membrane-associated genes, suggesting the key regulatory role of this gene in maintaining bacterial membrane homeostasis. A previous study revealed that SppA in *E. coli* cleaves signaling peptides released or extracted from membranes and performs a quality-assurance role for periplasmic and membrane-bound proteins (47). In addition, accumulation of these signaling peptides in the periplasm of the cell potentially interferes with protein translocation and affects cell membrane integrity through the Sec machinery (48, 49). For example, studies in *B. subtilis* have shown that *sppA* knockout strains have significantly reduced levels of protein substrates secretion, a result that directly confirms the important role of the *sppA* gene in the protein secretion process in this bacterium (16). Our study revealed that the Δ*sppA* mutant strain exhibited significant upregulation of an ECF-type sigma factor (AlgU; log2FC = 1.64) and the stress response protein OsmC (log2FC = 1.20). Previous study found that *osmC* upregulation may assist in protein refolding under membrane stress conditions (50), which promotes clearance of accumulated periplasmic proteins. Furthermore, upregulation of the MacAB-TolC efflux pump may help bacteria excrete signal peptide fragments or other stress-related toxic molecules, thereby relieving pressure on the membrane system (51). Since the pump efflux system was activated, we would like to know if the antibiotic resistance (AMR) is altered in the mutant strain. Surprisingly, the susceptibility of the mutant was not changed using the common recommended antibiotics in aquaculture (e.g., oxytetracycline, enrofloxacin and florfenicol). Oxytetracycline and florfenicol function by inhibiting protein synthesis via binding reversibly to the 30S and 50S ribosomal subunits, respectively. Enrofloxacin is a bactericidal agent that targets DNA gyrase and topoisomerase IV, thereby inhibiting DNA replication. This indicates that the deletion of *sppA* did not substantially compromise the membrane integrity, and the activated efflux pump activity did not change the tested antimicrobial susceptibility. The lack of altered antibiotic susceptibility suggests that the upregulated efflux system is specifically involved in exporting accumulated peptides or stress-related substrates to mitigate cellular toxicity, rather than functioning as a general efflux pump for the tested antibiotics.

The Δ*sppA* mutant strain exhibited a significant increase in OMV production, which was consistent with the changes in expression of several membrane-associated genes in the transcriptomic data. TEM analysis revealed a 3.8-fold increase (*p <* 0.0001) in OMV production. These findings were consistent with the mechanism reported by MacDonald and Kuehn (40), where the *algU σ* factor alleviates bacterial stress by promoting OMV formation, demonstrating a conserved membrane stress response strategy among Gram-negative bacteria. Previous report revealed that OMV biogenesis is an adaptive mechanism for membrane stress, whose activation can help eliminate accumulated misfolded proteins (52). Consistent with their study, we speculate that under the proteotoxic stress caused by *sppA* deletion, *F. columnare* compensates for the substrate accumulation by selectively packaging misfolded proteins into OMVs for extracellular disposal.

Signal peptide peptidases play an essential role in protein processing by degrading N-terminal signal peptides that have been cleaved from newly synthesized proteins. This degradation is a critical step for maintaining proper protein translocation and localization by preventing the accumulation of signal peptide fragments. The activity of signal peptide peptidases is potentially important for the function of the T9SS in *F. columnare*, which mediates the export of multiple virulence factors, including proteases and adhesins (6). Interestingly, based on our RNA-seq results, the transcription of T9SS-secreted proteins was not significantly changed in the Δ*sppA* mutant compared to the wild-type, suggesting that SppA does not regulate these proteins at the transcriptional level. Despite this, our findings indicate that the deletion of the *sppA* gene significantly reduces the virulence of *F. columnare* in freshwater Medaka fish under the tested conditions. This could possibly be due to the disrupted protein secretion system and elevated membrane stress in the knockout mutant. The accumulation of uncleared signal peptides in the Δ*sppA* could potentially induce membrane stress or hinder the protein translocation machinery. Consequently, although T9SS-secreted proteins are still synthesized normally, their efficient export or proper assembly might be compromised. This potential impairment could also explain the observed defects in biofilm formation. Biofilm development is a highly pleiotropic physiological process. Therefore, while the impaired gliding motility plays a major role, the potential failure to properly secrete T9SS-dependent adhesins, combined with general membrane stress and overall growth defects caused by *sppA* deletion, likely exerts a comprehensive negative impact on the cell’s ability to form biofilms. Additionally, a previous study found that σE/AlgU activation inhibited the expression of virulence genes as a protective response (53). This trade-off causes bacteria to prioritize maintaining cellular homeostasis over virulence factor production under membrane stress conditions, resulting in a partial reduction in virulence. While the mechanisms underlying this potential secretion impairment require further experimental validation, our result demonstrates that though the attenuation is moderate, *sppA* contributes to *F. columnare* pathogenicity and functions as a necessary virulence factor.

In conclusion, our findings provide new insights into the biological roles of *sppA* in the aquatic pathogen *F. columnare*. Our results reveal that *sppA* is essential for maintaining membrane homeostasis and normal cellular physiology, and the partial reduction in virulence indicates that *sppA* plays an important role in *F. columnare* pathogenesis. These results identified SppA as a potential target for developing strategies to manage columnaris disease in aquaculture.

## Data Availability

The data presented in the study are deposited in the NCBI Sequence Read Archive (SRA) repository, under BioProject accession number PRJNA1321558.
