# Supplementary material for: Pleiotropic effects of signal peptide peptidase A (sppA) gene deletion on membrane homeostasis, gliding motility, and virulence in Flavobacterium columnare
Source: Front Vet Sci. 2026 May 22;13:1784124. doi: 10.3389/fvets.2026.1784124 (PMC13236525; doi:10.3389/fvets.2026.1784124)
Supplement: Supplementary file 2 [file Data_Sheet_1.docx]

**Supplemental Figure, Movie Legend, and Table**

**Pleiotropic effects of signal peptide peptidase A (*sppA*) gene deletion on membrane homeostasis, gliding motility, and virulence in *Flavobacterium columnare***

**Authors:** Ruoxi Zhu, Liang Zhong, Yuying Xun, Shucheng Zheng, Yongtao Zhu, and Wenlong Cai

**Supplemental Figure**

**
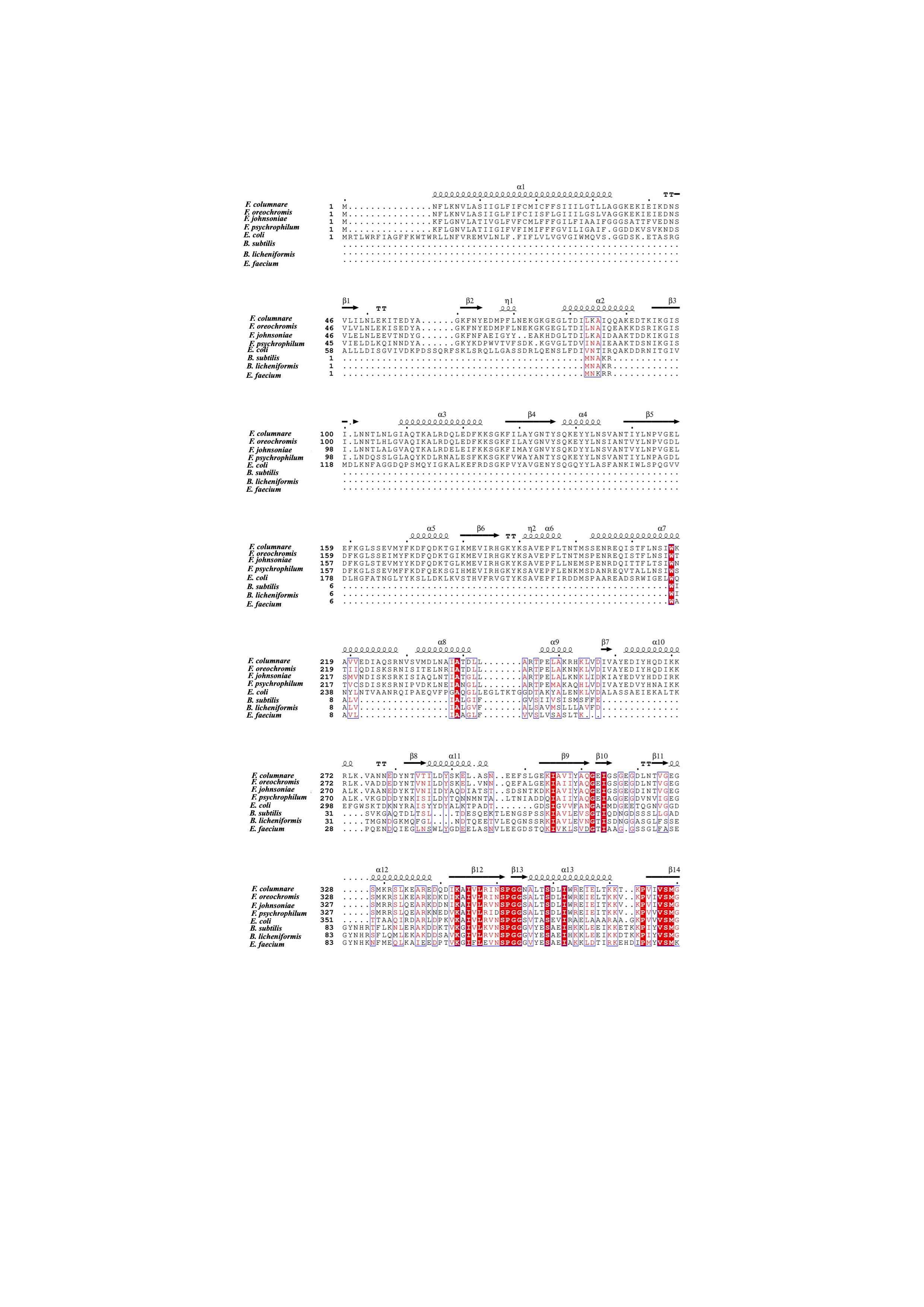
**

**
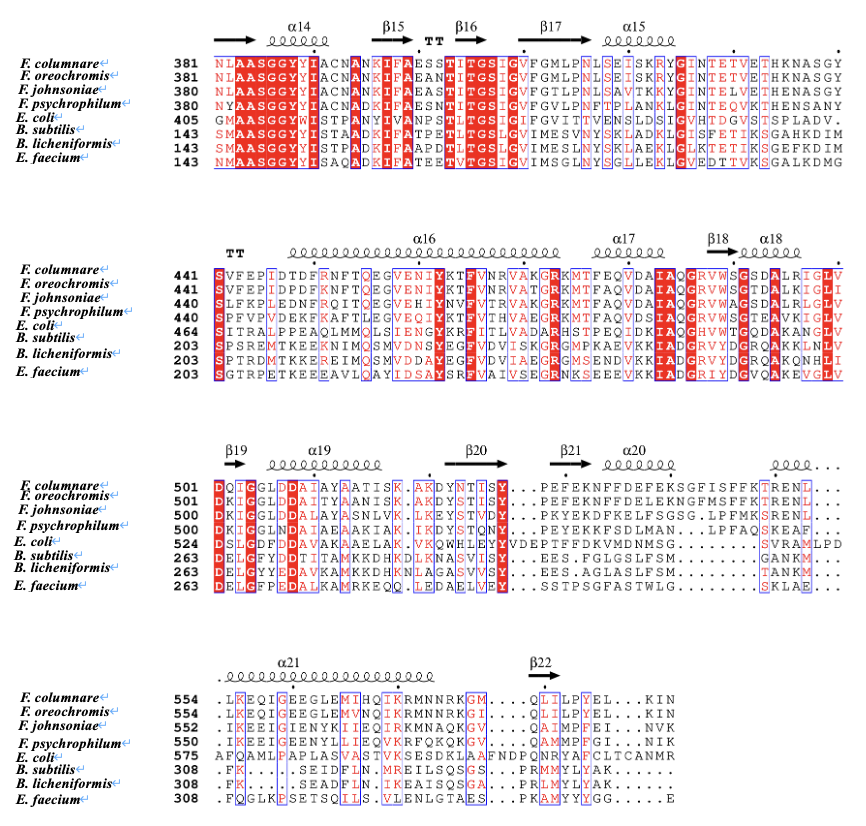
**

**Figure S1. Multiple sequence alignment and secondary-structure annotation of SppA homologues.** Alignments were performed using MAFFT and Espript 3.2 **(**[**https://mafft.cbrc.jp/alignment/server/index.html**](https://mafft.cbrc.jp/alignment/server/index.html)**;** [**https://espript.ibcp.fr/ESPript/ESPript/index.php**](https://espript.ibcp.fr/ESPript/ESPript/index.php)**).** Amino acid sequence alignment of SppA from *F. oreochromis* (WP_088398786), *F. psychrophilum* (BHD32194), *F. johnsoniae* (WP_012023794), *E. coli* (BAA15557), *B. subtilis* (CAB14931), *B. licheniformis* (WP_003184383), *E. faecium* (WP_002294607), with *F. columnare* (XUP20605). Identical residues are represented on a red background, and residues that are conserved across groups are boxed in blue. The secondary structures of SppA are depicted on top.

**
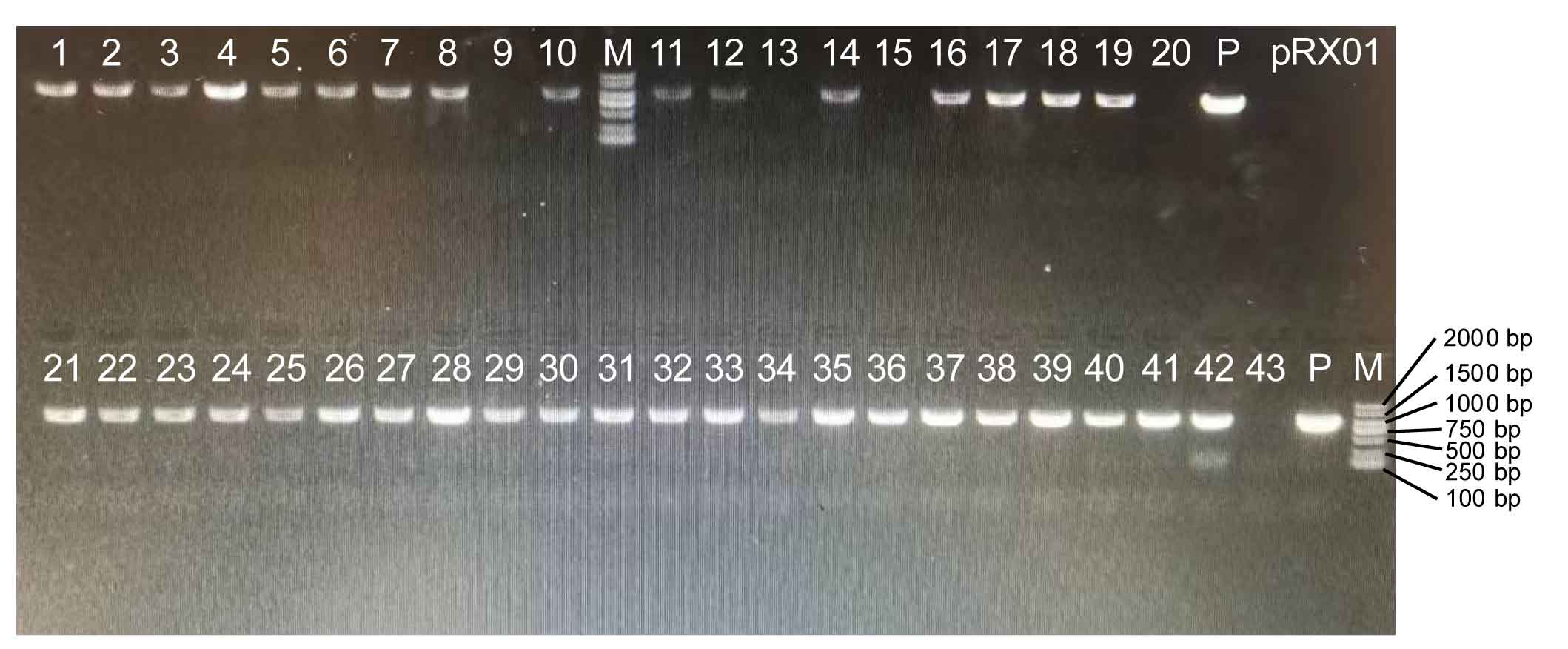
**

**Figure S2. Original agarose gel image corresponding to Figure 2B.**

This gel shows the PCR verification of the *sppA* gene deletion in *F. columnare* strains. Lanes 1–43 represent individual transformants screened using the primer pair S-L and S-R, which are located within the deleted region of the *sppA* gene. Lane M contains the DNA ladder (100 bp to 2000 bp), and lane P contains wild-type genomic DNA used as the PCR template, serving as the wild-type control. Lanes 13, 15, and 20 in this original image correspond to lanes 3, 5, and 10, respectively, in Figure 2B, and were selected as representative clones for downstream validation. A ~993 bp band indicates the presence of the intact *sppA* gene (wild-type), whereas the absence of a band (0 bp) indicates successful deletion of the *sppA* gene. Lane pRX01 represents the recombinant plasmid used as the PCR template. As expected, no band is observed in lane pRX01, confirming that the plasmid lacks the *sppA* sequence targeted by the S-L/S-R primers, and is therefore consistent with the deletion genotype.


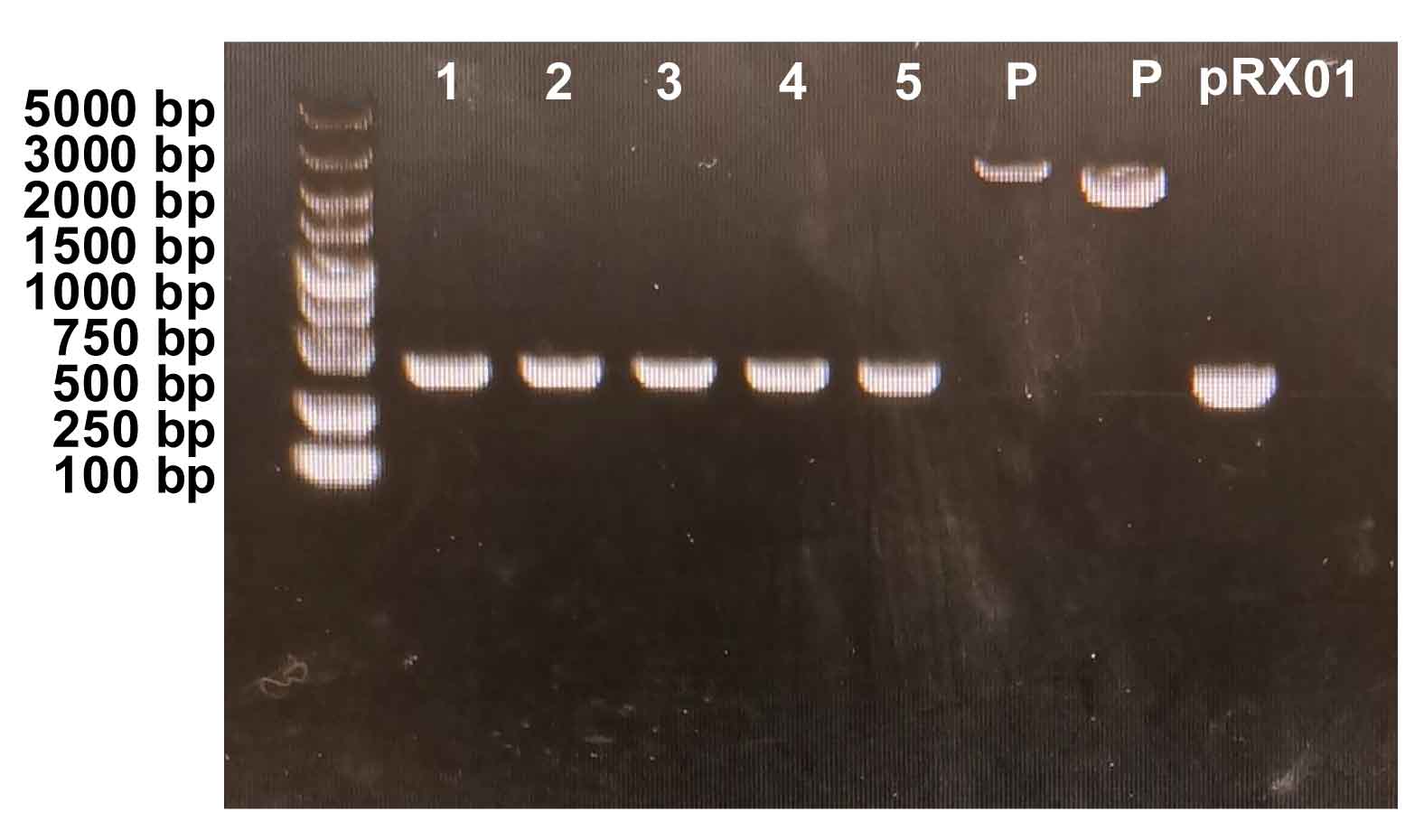


**Figure S3. Original agarose gel image corresponding to Figure 2C.**

This gel shows the PCR confirmation of the *sppA* gene deletion mutants in *F. columnare* using primers S-LEFT and S-RIGHT, which were designed within the upstream and downstream sequences of the deleted region. As a result, the amplicons from deletion mutants are significantly shorter than those from the wild-type strain due to the absence of the 1680 bp target region. Lanes 1, 2, and 3 represent three independent Δ*sppA* mutants, which correspond to lanes 1, 2, and 3 in Figure 2C, respectively. Lanes 4 and 5 are additional Δ*sppA* candidates. Lane P contains wild-type genomic DNA and shows a ~2040 bp band, consistent with the full-length *sppA* region. Lane pRX01 contains recombinant plasmid pRX01 used as the template; the smaller ~360 bp band confirms the deletion construct.

**Supplemental Movie Legend**

**Movie S1.** Bacterial gliding motility (WT, Δ*sppA* mutant, and complementary strains) at the single-cell level under time-lapse microscopy. Cells were added to glass tunnel slides, incubated for 5 min, and cell motility was recorded at 25°C using a Nikon Ci-L plus microscope equipped with an SC2000C CMOS camera. Three 30-second sequences are shown. Bars indicate 10 µm. The initial frames and rainbow traces in Figure 4 of the main text were derived from these movies.

**Table S1. The minimum inhibitory concentrations of the wild-type strain and the *sppA* gene deletion mutant.** The antibiotic resistance to OTC in the C-*sppA* strain was possibly due to the anti-TET gene introduced in the pCP23 plasmid.

| Antibiotic | MIC ($\mu$g/mL) | | |
| --- | --- | --- | --- |
|  | wild type | *ΔsppA* | C-*sppA* |
| Oxytetracycline dihydrate (OTC) | 0.25 | 0.25 | 8 |
| Florfenicol (FF) | 1 | 1 | 1 |
| Enrofloxacin (ENRO) | 0.03 | 0.03 | 0.03 |
